# 多原发性肺癌的诊治进展

**DOI:** 10.3779/j.issn.1009-3419.2015.10.07

**Published:** 2015-10-20

**Authors:** 汶鑫 罗, 萍 周, 为民 李

**Affiliations:** 610041 成都，四川大学华西医院呼吸科 Department of Respiratory, West-China Hospital of Sichuan University, Chengdu 610041, China

**Keywords:** 肺肿瘤, 分子标志物, 突变, 手术, 立体定向放疗, Lung neoplasms, Molecular markers, Mutations, Surgery, Stereotactic ablative radiotherapy

## Abstract

多原发性肺癌的发病率和检出率逐年升高。目前临床上诊断多原发性肺癌(multiple primary lung cancer, MPLC)主要参照Martini-Melamed标准和美国胸科医师协会(American College of Chest Physicians, ACCP)标准, 综合考虑临床表现、影像学特征、组织学类型和分子遗传学特征。组织学类型不同的MPLC诊断相对容易, 而组织学类型相同的MPLC诊断仍相当困难。DNA倍体分析、基因突变检测、微卫星多态性分析等分子生物学技术为MPLC的正确诊断提供了新手段, 可评估各病灶的克隆性关系, 帮助鉴别MPLC与转移。MPLC的首选治疗方案为根治性手术, 术式应考虑患者肺功能储备等因素, 选择肺叶切除、肺段切除或楔形切除; 对于不能根治性切除的病灶, 可综合化疗、放疗、立体定向放疗(stereotactic ablative radiotherapy, SABR)、射频消融(radiofrequency ablation, RFA)、分子靶向治疗等。

## Abstract (en)

Recently, the incidence and detection rates of multiple primary lung cancer (MPLC) are increasing.The diagnosis of MPLC depends mainly on the Martini-Melamed criterion and ACCP criterion at present, taking all features (histological, genetic, radiologic and clinical) into account.It may be easy to diagnose cases of MPLC that exhibit different histological types, but it is difficult to diagnose cases that exhibit similar histological type.DNA polity, gene mutations, microsatellite alteration and so on provide new methods for the accurate diagnosis of MPLC.They can evaluate the clonal relationship and help differential diagnosis between MPLC and metastasis.The first therapeutic choice for MPLC is curative operation.The surgical approach includes lobectomy, wedge resection and segmentectomy.For those which cannot be resected, we can synthesize chemotherapy, radiotherapy, stereotactic ablative radiotherapy (SABR), radiofrequency ablation (RFA), molecular targeted therapy, *etc*.

多原发性肺癌(multiple primary lung cancer, MPLC)是指在同一患者肺内不同部位同时或先后发生两个或两个以上原发病灶的肺癌。根据各原发病灶发生的时间关系, 可将MPLC分为同时MPLC(synchronous MPLC, SMPLC)和异时MPLC(metachronous MPLC, MMPLC)。1924年, Beyreuther^[1]^首先在尸检中发现了1例双侧MPLC合并肺结核的病例。随着诊断技术的进步[如高分辨计算机断层扫描(computed tomography, CT)、正电子发射型计算机断层显像(positron emission computed tomography, PET)-CT、纤支镜等]、低剂量CT筛查肺癌的广泛应用、人口老龄化以及临床医师对MPLC的重视, MPLC的发病率和检出率逐年升高。因此, 如何正确诊断与合理治疗MPLC是我们急需解决的临床问题。本文就近年来MPLC诊断治疗方面的研究进展作一综述。

## 1 MPLC的诊断

1975年, Martini和Melamed^[2]^建立了MPLC的临床病理诊断标准。包括:SMPLC:①病灶部位不同, 相互独立; ②组织学类型不同; ③组织学类型相同, 但位于不同的肺段、肺叶或双侧肺, 且起源于不同的原位癌, 共同的淋巴引流部位无癌, 确立诊断时无肺外转移。MMPLC:①组织学类型不同; ②织学类型相同时, 满足以下任意1条:a.无瘤间隔期≥2年, b.起源于不同的原位癌, c.再发原发癌位于不同肺叶或对侧肺, 且共同的淋巴引流部位无癌, 确立诊断时无肺外转移。该标准至今仍被广泛应用, 强调各病灶具有不同的病理形态特征。但当各病灶组织学类型相同时, 鉴别MPLC与转移存在困难, 因为很难在治疗前了解各病灶是否来源于不同的原位癌或共同的淋巴引流部位是否有癌。1995年, Antakli等^[3]^对Martini-Melamed标准做了补充, 提出进行DNA倍体分析来帮助鉴别MPLC与转移, 但未提出可靠的技术手段来进行基因分析。

2003年, 美国胸科医师协会(American College of Chest Physicians, ACCP)推荐了新的MPLC诊断标准^[4]^, 并于2007年^[5]^和2013年^[6]^做出了更新。包括:SMPLC:①组织学类型不同, 分子遗传学特征不同或起源于不同的原位癌; ②组织学类型相同时, 癌肿位于不同的肺叶, 无N2、N3转移且无全身转移。MMPLC:①组织学类型不同, 分子遗传学特征不同或起源于不同的原位癌; ②组织学类型相同时, 无瘤间期≥4年, 无全身转移。提出可采用特异的分子标志物或基因点突变检测来分析分子遗传学特征; 也可通过肺腺癌组织学亚型(如鳞屑样、乳头状、腺泡样等)加以鉴别。强调MPLC的诊断应由放射科、呼吸内科、胸外科及病理科医生共同参与, 综合考虑临床表现、影像学特征、组织学类型和分子遗传学特征。

在MPLC的诊断中, 其影像学表现具有重要意义。对于确诊或高度疑诊原发性肺癌合并有其他可疑肺癌结节的患者, 我们应仔细分析其影像学特征。研究结果显示, 与实性结节相比, 亚实性结节的恶性概率高, 而部分实性结节的恶性概率较单纯磨玻璃结节高^[7]^。不论是实性结节还是亚实性结节, 直径小于8 mm的亚厘米结节的恶性程度均偏低^[8]^。与边界光滑的肺结节相比, 边缘有毛刺或边界不规则的肺结节的恶性概率增加5倍; 具有胸膜凹陷征的肺结节的恶性概率增加1倍; 血管征和分叶状则分别使恶性几率增加70%和10%^[9]^。

## 2 分子生物学研究

诊断组织学类型不同的MPLC相对容易, 但诊断组织学类型相同的MPLC仍相当困难。然而, 明确诊断很大程度上决定治疗方案并影响预后。因此, 临床上鉴别多发肺癌病灶是MPLC还是转移十分重要。近年来, DNA倍体分析, *p53*基因、表皮生长因子受体(epidermal growth factor receptor, *EGFR*)基因和*Kras*基因突变检测以及微卫星多态性分析应用于多发肺癌病灶的基因比较分析, 评估各病灶的克隆关系, 鉴别MPLC与转移。

Arai等^[10]^利用微阵列比较基因组杂交技术(array comparative genomic hybridization, aCGH)检测了12例组织学类型相同的双肺癌病灶患者的基因拷贝数变化。发现基因拷贝数变化一致率在经临床诊断的6例肺内转移患者中明显高于6例双发原发性肺癌患者; 将50%的一致率设定为诊断界限, 结果显示10例aCGH基因分析与临床诊断一致, 另外2例不一致, 但通过详细的临床病理分析也支持aCGH基因分析结果。Girard等^[11]^做了类似的研究, 发现22组配对肺癌病灶中有4组基因拷贝数变化分析与临床病理诊断不一致, 但通过*EGFR*/*Kras*基因突变检测也支持基因拷贝数变化分析结果。因此, 推荐联合基因拷贝数变化分析/基因突变分析和临床病理分析来鉴别MPLC和转移。

Girard等^[12]^研究了7例双发肺腺癌病灶患者的*EGFR*/*Kras*基因突变。发现根据Martini-Melamed标准^[2]^, 6例为多原发性肺腺癌, 1例为转移; 根据ACCP标准^[6]^, 3例为多原发性肺腺瘤, 3例为转移, 1例无法分类; 而基因突变检测提示所有患者配对肿瘤的*EGFR*/*Kras*基因突变均不同, 即为双发原发性肿瘤, 且与肺腺癌组织学亚型的结论一致。提示分别参照Martini-Melamed标准和ACCP标准通过临床病理分析诊断组织学类型相同的MPLC, 可能得出不一致的结果。而*EGFR*/*Kras*基因突变分析可鉴别MPLC和转移, 更好地完善目前使用的临床病理诊断标准。

Shen等^[13]^研究了5例组织学类型不同的SMPLC、8例组织学类型相同的SMPLC和10例转移性肺癌患者的6个微卫星标记(2q34、6q23.2、7q33、10q24.3、15q12和22q12.1)的等位基因变异。发现10例转移性肺癌患者各病灶的微卫星标记的等位基因变异模式均一致; 组织学类型不同的5例SMPLC患者均不一致; 而组织学类型相同的8例MPLC患者中, 2例一致, 6例不一致。提示微卫星变异分析可评估多发肺癌病灶的克隆关系, 有助于鉴别诊断MPLC和转移。

Ono等^[14]^研究了*p53*、*p16*、*p27*及*c-erbB2*四个癌症相关基因在50例组织学类型相同的MPLC、20例肺内转移、11例组织学类型不同的MPLC及30例淋巴结转移患者中的表达, 结果发现淋巴结转移组中总差异表达阳性率均未超过90%, 组织学类型不同的MPLC组中总差异阳性表达率为110%-220%, 均大于90%。进一步将90%作为诊断临界值, 总差异阳性表达率大于90%时, 认为这两个肺癌病灶是相互独立的原发病灶, 即MPLC, 结果显示18%(9/50)的组织学类型相同的MPLC患者(依据Martini-Melamed标准)总差异阳性表达率未超过90%, 被重新诊断为肺内转移; 20%(4/20)的肺内转移患者总差异阳性表达率大于90%, 被重新诊断为MPLC。Chen等^[15]^对36例经临床诊断的组织学类型相同的MPLC、20例肺内转移、14例组织学类型不同的MPLC、30例淋巴结转移及11例远处转移患者进行了类似的研究, 获得了相同结果, 19.4%(7/36)的组织学类型相同的MPLC患者被重新诊断为肺内转移, 30%(6/20)的肺内转移患者被重新诊断为MPLC。因此, 完全根据目前广泛使用的临床诊断标准来鉴别组织学类型相同的多发肺癌病灶存在一定困难, 通过联合检测癌症相关基因在多发肺癌病灶中的差异表达有助于鉴别MPLC和肺内转移, 并可用于肺癌分期, 且此方法假阴性率低^[14]^。

## 3 治疗进展

MPLC各病灶是相互独立的, 应对各病灶分别进行肿瘤-淋巴结-转移(tumor-node-metastasis, TNM)临床分期, 争取早期积极治疗。无纵隔淋巴结转移及全身转移的临床证据且心肺功能和全身状况允许时, 应争取根治性手术^[6]^。对于不能手术的病灶, 可综合化疗、放疗、立体定向放疗(stereotactic ablative radiotherapy, SABR)、射频消融(radiofrequency ablation, RFA)、分子靶向治疗等。

手术术式的选择需考虑术者的操作技巧、患者的心肺功能等情况, 遵循最大限度切除肿瘤和最大限度保留正常肺组织的原则。Toufektzian等^[16]^研究发现肺叶切除及亚肺叶切除(肺段切除或楔形切除)预后较全肺切除好; 肺段切除与楔形切除预后无差异。综上, SMPLC位于同一肺叶时, 可行单一肺叶切除; SMPLC位于单侧不同肺叶或双侧肺时, 术前充分评估患者肺功能储备等情况, 结合肺叶、肺段和楔形切除是必要的。MMPLC应积极选择肺叶切除, 而肺功能储备不足时, 仍建议行肺段或楔形切除。

SABR是一种新兴的放射治疗方法, 与传统放疗相比, SABR能够延长患者生存, 并减少放疗毒副反应的发生^[17]^。Chang等^[18]^回顾性分析了101例因无法耐受手术而行SABR的早期MPLC患者的预后, 2年及4年局部控制率分别为97.4%、95.7%, 2年及4年总生存率分别为73.2%、47.5%, 2年及4年无复发生存率分别为67.0%、58.0%。Creach等^[19]^研究了行SABR的66个MPLC癌肿的预后, 发现中位无复发生存期及中位总生存期分别为15.5个月、20个月, 仅6例癌肿放疗处有复发, 均无3级以上毒副反应发生。提示SABR对于MPLC可取得较好的预后, 对于无法耐受手术的早期MPLC是一种安全有效的治疗方式。

RFA作为一种微创治疗方法, 近年来在治疗肺部肿瘤方面已取得了理想疗效^[20]^, 特别是直径 < 3 cm的周围型Ⅰ期(non-small cell lung cancer, NSCLC)^[21]^。Dupuy等^[22]^对55例行RFA的Ⅰa期NSCLC患者进行了多中心前瞻性研究, 结果显示1年及2年总生存率分别为86.3%和69.8%, 1年及2年局部控制率分别为68.9%和59.8%, 12例患者发生3级以下毒副反应。提示CT引导下经皮肺穿刺射频消融治疗不能手术的早期NSCLC安全、有效, 可成为不能手术的早期MPLC又一种可选择的治疗手段。

*EGFR*和间变淋巴瘤激酶(anaplastic lymphoma kinase, *ALK*)这两个基因靶点的突变检测和相关药物的发展, 使无法手术的NSCLC有了新的治疗手段—分子靶向治疗。已有多项临床研究^[23-26]^证明, EGFR酪氨酸激酶抑制剂(tyrosine kinase inhibitor, TKI)和ALK抑制剂能分别使*EGFR*基因敏感突变和*ALK*融合基因阳性的NSCLC患者获益。对于不能手术的MPLC, 我们可通过穿刺等方法取得标本进行基因突变检测, 使分子靶向治疗成为其另一种可选择的治疗手段。

## 4 展望

目前国内外仍缺乏对MPLC诊断标准、分子生物学指标及治疗选择的共识或指南。大量研究表明DNA倍体分析、基因突变检测、微卫星多态性分析等分子生物学技术有助于MPLC的诊断及鉴别诊断, 但仍缺乏多中心大样本的临床研究证实。SABR、RFA、分子靶向治疗等为MPLC的治疗带来了新的选择, 但其对MPLC的治疗价值如何需进一步研究。
